# Centralised Identification of Early‐Gestation Biospecimens to Support Early‐Onset Pre‐Eclampsia Prediction

**DOI:** 10.1111/1471-0528.70196

**Published:** 2026-02-20

**Authors:** Chris E. Philip, Hani Faysal, Sara K. Quinney, Shaohong Feng, David M. Haas

**Affiliations:** ^1^ Department of Obstetrics and Gynecology Indiana University School of Medicine Indianapolis Indiana USA; ^2^ Department of Obstetrics and Gynecology University of Texas Southwestern Medical Center Dallas Texas USA; ^3^ Department of Biomedical Informatics The Ohio State University Wexner Medical Center Columbus Ohio USA

**Keywords:** biobanking, biospecimen, early‐onset preeclampsia, health services research, medical disorders in pregnancy

The Collaborative Online Perinatal & Paediatric Repository (COPPER), supported by the NICHD Maternal and Paediatric Precision in Therapeutics (MPRINT) initiative, functions as a centralised directory of pregnancy and paediatric biobanks. While COPPER does not store or broker biospecimens, it offers a streamlined way for researchers to locate relevant biospecimen sources for maternal and child health studies. Biobanks in the directory include a wide range of sample types such as cord blood, breast milk, urine, placental tissue and blood derivatives (plasma and serum), enabling a broad spectrum of research inquiries. This feasibility exercise aimed to evaluate whether a hypothetical study to evaluate biomarkers for early‐onset preeclampsia with severe features could be facilitated by the COPPER database. The objective was to use the database to identify the number of qualifying biospecimens across COPPER‐linked repositories.

We contacted coordinators of the 17 current COPPER biobanks and requested information on available biospecimens from participants who (1) had plasma or serum collected before 16 weeks' gestation (≥ 100 μL per aliquots) and (2) were diagnosed with early‐onset preeclampsia with severe features (defined as diagnosed at ≤ 32 weeks of gestation or delivered by that time). Chronic hypertension at baseline was an exclusion criterion. A stipend was offered to offset staff time for data retrieval. No physical specimens or participant specific information were requested, only information on the number of samples that could be available and basic cohort characteristics.

Of the 17 biobanks contacted, 10 did not meet eligibility criteria based on specimen type or clinical characteristics. Among the remaining 7, five (71%) provided data. One biobank declined the stipend, and one reported limited sample availability due to prior use in another project.

In total, up to 112 unique cases of early‐onset pre‐eclampsia with early gestation biospecimens were identified across the five responding biobanks. All specimens were collected < 16 weeks' gestation. Nearly equal amounts of plasma and serum samples could be available. Only one of the responding biobanks was NIH funded and in the NICHD Data and Specimen Hub (DASH). The majority of biobanks that would contribute are thus not part of other governmental registries and would be difficult to identify otherwise.

Our feasibility assessment demonstrated that most qualifying biobanks within the COPPER directory were responsive and willing to share aggregate biospecimen data. COPPER provides a centralised mechanism to identify early gestational biospecimens across multiple independent repositories, enabling aggregation of samples that would be underpowered in single centred studies. For relatively uncommon conditions such as early‐onset preeclampsia with severe features, individual studies often lack sufficient early pregnancy biospecimens, highlighting the need for collaborative approaches. Centralised aggregation of early gestation biospecimens that may reside in different biorepositories can aid investigators to overcome sample size limitations, thus supporting biomarker discovery and validation for precise medicine approaches in preeclampsia and other conditions. In this exploration, the identification of up to 112 unique participants with early‐gestation plasma or serum samples provides a meaningful foundation for early prediction and risk stratification research, particularly when paired with available contemporaneous normotensive controls. Although not sufficient for definitive model development, aggregation of this magnitude is well suited for biomarker discovery, external validation and feasibility assessments that inform future interventional trial design. While identifying 112 cases may not seem like large numbers, considering that a recent study from Spain (Castillo‐Marco et al., *Nature Communications*, 2025) needed to recruit almost 9600 participants and only ended up with 42 cases of preeclampsia at < 34 weeks, the number of specimens identified is a more efficient means of performing these types of studies. These results support COPPER's value in connecting researchers to biospecimens and data, thereby accelerating perinatal and paediatric translational research. Expansion of centralised biobank directories to include international repositories could further enhance sample diversity, generalizability and global collaboration in perinatal precision‐medicine research. While we did not explore variability in data collected by the different COPPER biobanks for these cases, future work is focusing on how to harmonise consent language for specimen sharing and metadata consistency. For investigators who have available perinatal or child data and/or specimens, please consider adding your information to this resource at https://copper.osubmi.org/submit.

## Author Contributions

C.E.P. and D.M.H. conceived the study, coordinated data collection and drafted the manuscript. H.F., S.F. and S.K.Q. contributed to data acquisition and interpretation. D.M.H. supervised the project and provided critical intellectual input. All authors reviewed and approved the final manuscript.

## Funding

This work was funded under National Institute of Child Health and Human Development (NICHD) Maternal and Paediatric Precision In Therapeutics (MPRINT) grant NIH P30HD10645. The content is solely the responsibility of the authors and does not necessarily represent the official views of the National Institutes of Health.

## Ethics Statement

The authors have nothing to report.

## Consent

The authors have nothing to report.

## Conflicts of Interest

The authors declare no conflicts of interest.

## Data Availability

The data that support the findings of this study are available from the corresponding author upon reasonable request.

